# COVID-19 and Alzheimer’s Disease Share Common Neurological and Ophthalmological Manifestations: A Bidirectional Risk in the Post-Pandemic Future

**DOI:** 10.3390/cells12222601

**Published:** 2023-11-10

**Authors:** Giuseppina Amadoro, Valentina Latina, Egidio Stigliano, Alessandra Micera

**Affiliations:** 1 Institute of Translational Pharmacology (IFT), National Research Council (CNR), Via Fosso del Cavaliere 100, 00133 Rome, Italy; v.latina@ebri.it; 2 European Brain Research Institute (EBRI), Viale Regina Elena 295, 00161 Rome, Italy; 3 Area of Pathology, Department of Woman and Child Health and Public Health, Fondazione Policlinico Universitario A. Gemelli IRCCS, Istituto di Anatomia Patologica, Università Cattolica del Sacro Cuore, Largo Francesco Vito 1, 00168 Rome, Italy; egidio.stigliano@policlinicogemelli.it; 4 Research and Development Laboratory for Biochemical, Molecular and Cellular Applications in Ophthalmological Sciences, IRCCS-Fondazione Bietti, Via Santo Stefano Rotondo, 6, 00184 Rome, Italy

**Keywords:** post-pandemic, COVID-19, Alzheimer’s Disease (AD), neurological disorders, brain, eyes

## Abstract

A growing body of evidence indicates that a neuropathological cross-talk takes place between the coronavirus disease 2019 (COVID-19) -the pandemic severe pneumonia that has had a tremendous impact on the global economy and health since three years after its outbreak in December 2019- and Alzheimer’s Disease (AD), the leading cause of dementia among human beings, reaching 139 million by the year 2050. Even though COVID-19 is a primary respiratory disease, its causative agent, the so-called Severe Acute Respiratory Syndrome coronavirus 2 (SARS-CoV-2), is also endowed with high neuro-invasive potential (Neurocovid). The neurological complications of COVID-19, resulting from the direct viral entry into the Central Nervous System (CNS) and/or indirect systemic inflammation and dysregulated activation of immune response, encompass memory decline and anosmia which are typically associated with AD symptomatology. In addition, patients diagnosed with AD are more vulnerable to SARS-CoV-2 infection and are inclined to more severe clinical outcomes. In the present review, we better elucidate the intimate connection between COVID-19 and AD by summarizing the involved risk factors/targets and the underlying biological mechanisms shared by these two disorders with a particular focus on the Angiotensin-Converting Enzyme 2 (ACE2) receptor, APOlipoprotein E (APOE), aging, neuroinflammation and cellular pathways associated with the Amyloid Precursor Protein (APP)/Amyloid beta (Aβ) and tau neuropathologies. Finally, the involvement of ophthalmological manifestations, including vitreo-retinal abnormalities and visual deficits, in both COVID-19 and AD are also discussed. Understanding the common physiopathological aspects linking COVID-19 and AD will pave the way to novel management and diagnostic/therapeutic approaches to cope with them in the post-pandemic future.

## 1. Introduction

The relationship between Alzheimer’s Disease (AD)—the most prevalent form of neurodegenerative dementia among the elderly with more than 55 million people worldwide [1,2]—and the global pandemic coronavirus disease 2019 (COVID-19), caused by the novel etiological agent known as Severe Acute Respiratory Syndrome coronavirus 2 (SARS-CoV-2), is receiving great attention from the scientific community due to its detrimental impact on healthcare and socioeconomic organizations [3]. 

Even though the most common clinical presentation of COVID-19 is interstitial pneumonia accompanied by a fever and gastrointestinal problems [4,5], a wide range of multisystemic/organ signs classified as “Post-Acute Sequelae of COVID-19 (PASC)”, “Long-term COVID-19 syndrome”, or colloquially “Long COVID”/”Long Haul” has been described in about 30% of affected patients, from 6 months up to 2 years after the initial phase of the viral infection [6,7,8,9,10]. Prolonged mental manifestations—including memory deficits and depression, confusion and anxiety accompanied by a marked sensory decline with a loss of taste and smell—frequently occur during the acute phase and/or the recovery period, as a part of the multi-faced and complex long COVID-19 syndrome. These epidemiological findings are in agreement with the capability of SARS-CoV-2 to penetrate the Central Nervous System (CNS) along multiple ways (neuroinvasion), where it can, directly and/or indirectly, infect both neurons and glial cells (neurotropism) and, possibly, induce and/or contribute to the development of neurological diseases (neurovirulence) [11,12]. This might not be surprising because the Angiotensin-Converting Enzyme 2 (ACE2) receptor for SARS-CoV-2 is widely distributed in different areas of the human brain, including the prefrontal cortex and hippocampus along with the ocular surface and associated structures [13]. Consistently, the neurotropism and the replication capacity of SARS-CoV-2 have been confirmed in neuronal cultures, brain organoids, mice and human brain autopsies [14,15,16,17,18]. Both viral-dependent (the viral invasion of brain parenchyma and vessels and/or replications) and viral-independent mechanisms (the hijacking of host innate immune response with inflammatory cytokine production, including perivascular inflammation) contribute to SARS-CoV-2-induced neuronal injuries and degeneration leading, eventually, to neurological and neuropsychiatric and neurosensorial symptoms [11,19].

AD is a chronic neurodegenerative disorder which is characterized by the progressive deterioration of cognitive functions due to the selective loss of vulnerable brain areas in association with olphactory and visual dysfunctions [20,21,22,23,24,25]. The extracellular senile plaques (SPs), mainly composed of the Amyloid-beta peptide (Aβ) aggregates, and the neurofibrillary tangles (NFTs), comprised of post-translational modified deposits of the intracellular microtubule-associated protein tau, are the two main distinctive histopathological lesions. Although the precise etiology is still unknown, aberrant protein misfolding, vascular damage involving large and small brain vessels, immunosenescence, neuroinflammation and the increased production of pro-inflammatory cytokines, Blood–Brain Barrier (BBB) breakdown, oxidative stress with the overproduction of Reactive Oxygen Species (ROS), mitochondrial dysfunction, synaptic derangement and inappropriate elimination and neural loss are implicated in the onset/progression of AD [20,21,22,23,24,25].

In this review, we highlight the clinical/epidemiological aspects and the molecular physiopathological mechanisms pointing to an increased susceptibility of developing AD in subjects that have experienced the COVID-19 infection. To support this finding, we give detailed insights into the neurochemical interplay occurring between COVID-19 and AD, both in the brain and eye, by paying particular attention to the many common risk factors and neuro-ophthalmological complications shared by these two disorders. We hope that this review, including for the first time a section devoted to ocular manifestations occurring both in COVID-19 and AD, will provide interesting information for researchers, clinicians and ophthalmologists working in the field. 

## 2. Neuroinvasive Mechanisms of SARS-CoV-2 and Neurological Manifestations of COVID-19 

**Figure 1:**
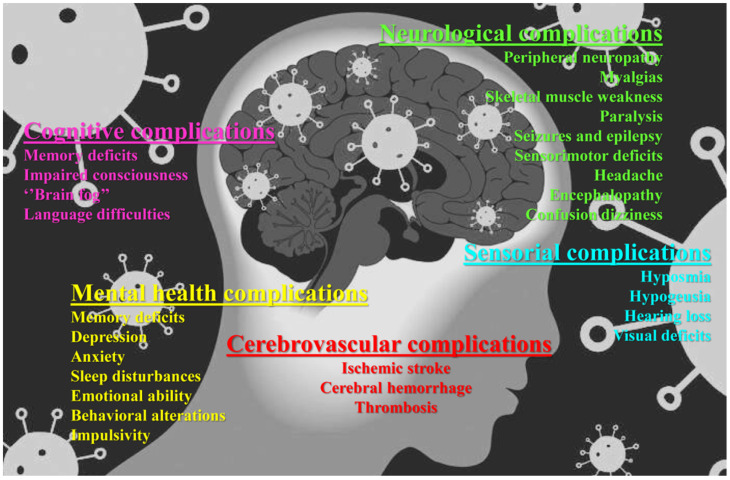
Long-term neurological and cognitive consequences of COVID-19. An illustration of the peripheral and central neurological manifestations of the post-COVID-19 syndrome.

Clinical and experimental evidence has shown that the SARS-CoV-2 infection can affect multiple organs beyond the respiratory system, including the Central (CNS) and Peripheral Nervous System (PNS), thus triggering *per se* neuronal injuries and/or exacerbating the neurodegenerative conditions of pre-existing diseases [26,27,28]. For mechanistic insights, the cerebral and mental complications of COVID-19 are the neuropathological consequences of one or a combination of all of the following factors: (1) direct viral neuronal damage leading to encephalitis (virus-induced neuropathology); (2) systemic inflammation with “cytokine storm” causing the damage of peripheral organs (the liver, kidney and lungs) which indirectly affects the brain’s health (neuroimmunopathology); (3) global ischemia secondary to respiratory insufficiency and the so-called acute respiratory distress syndrome (ARDS); and (4) cerebrovascular damage (blood vessels and coagulopathies) with ischemic or hemorrhagic strokes. Consistently, a high incidence of both CNS and PNS persistent symptoms and/or delayed or long-term neurological, sensorial and motor manifestations are associated with the pathogenesis of SARS-CoV-2 infection. Hyposmia, headache, dizziness, ataxia, cerebrovascular injury, hypogeusia, nausea, encephalitis, fatigue, myalgia, ataxia, neuropathies, conjunctivitis, retinopathy, encephalopathy, myelitis, vomiting, delirium, psychosis, ischemic stroke, epileptic seizures, neurocognitive and psychiatric complications, acute respiratory distress syndrome and affective disorders are recorded in observational studies on COVID-19 survivors [26,29,30,31] (Figure 1). More importantly, the SARS-CoV-2 neuroinvasion of the CNS has been claimed based on quite a few in vitro and in vivo analyses ranging from immunohistochemistry, in situ hybridization, Real-Time Polymerase Chain Reaction (RT-PCR) and Transmission Electron Microscopy (TEM) carried out on autoptic brain tissues and the CerebroSpinal Fluid (CSF) of patients who died of COVID-19 to experimental evidence on human-induced Pluripotent Stem Cells (iPSCs) and brain organoids and animal models [11,17,32,33,34,35,36,37,38]. However, it is also worth noting that, up to now, the definite evidence of SARS-CoV-2’s presence in the nervous system is a matter of debate since neither viral RNA nor particles have been found in human tissues and CSFs by other researchers [39,40,41,42,43]. Moreover, not all animals inoculated with the SARS-CoV-2 virus have shown neurological complications or full-blown CNS infections [44,45]. 

**Figure 2:**
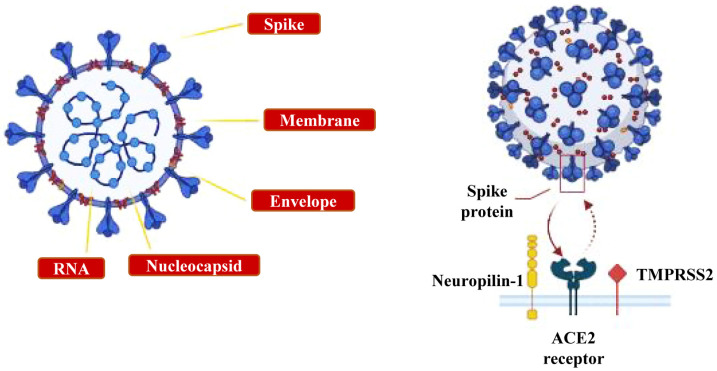
SARS-CoV-2 virus and its receptors. An illustration showing the SARS-CoV-2 structure and its membrane receptors. This figure was created with BioRender.com.

In general, the replication process of SARS-CoV-2 into host cells requires the initial binding of viral Spike Protein 1 (SP1) to its membrane-anchored ACE2 receptor, even though other proteins such as integrins, neuropilin-1 and the TransMembrane PRoteaSes Serine 2 and Serine 4 (TMPRSS2 and TMPRSS4, respectively) can also take part in it [27,46,47,48,49] (Figure 2). Relevantly, the evidence that the ACE2 receptor as long as two other co-receptors such as TMPRSS2 and neuropilin-1 [27] are widely distributed throughout the CNS and PNS, including the brainstem, cortex, striatum, hypothalamus, choroid plexuses, spinal cord, olfactory neuroepithelium, retinal ganglion cells, tongue gustatory nerve and neuromuscular junction [26,27,28], provides the strong biological rationale for SARS-CoV-2 neurotropism [31,32,50]. 

**Figure 3:**
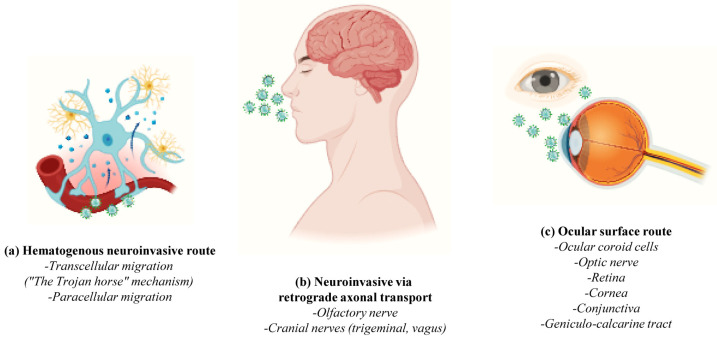
Neuroinvasive mechanisms of SARS-CoV-2. A picture showing the different routes through which SARS-CoV-2 enters the brain. This figure was created with BioRender.com.

In this connection, several both direct and/or indirect routes for the invasion of SARS-CoV-2 into the nervous system (Figure 3) have been proposed [26,30,51,52,53]:

- The hematogenous pathway or “Trojan horse mechanism” wherein infected circulating immune cells serve as reservoirs for the virus that traverses from the bloodstream to the CNS (cell transmigration); SARS-CoV-2 infects, near the vessel wall, the resident peripheral immune cells of the blood circulation (phagocytic monocytes/macrophages, neutrophils and lymphocytes) which, in turn, penetrate the neurovascular unit of the Blood–Brain Barrier (BBB), becoming a pool of viral dissemination toward the CNS [54,55,56] (Figure 3a);
- The Blood–CerebroSpinal Fluid (B-CSF) pathway (paracellular migration): SARS-CoV-2 binds to the ACE2 receptors of the endothelial cells and damages the integral citoarchitecture of the BBB. To get into the brain, the virus locally activates the signaling transduction pathway of Nuclear Factor kappa B (NF-kB) transcription factor, leading to an up-regulation in the basal expression level of Matrix MetalloPeptidase 9 (MMP9) which, in turn, degrades the extracellular matrix with consequent increased B-CSF permeability and alterations in immune cell trafficking (MMP8, Monocyte Chemoattractant Protein-1 (MCP-1), InterCellular Adhesion Molecule 1 (ICAM-1), a neuroinflammatory response with the release of pro-inflammatory cytokines and chemokines such as Interleukin (IL) IL-2, IL-6, IL-7 and IL-8, Tumor Necrosis Factor (TNF) TNFα, C-C Motif Chemokine Ligand (CCL) CCL2, CCL3 and CCL7 and C-X-C motif chemokine ligand (CXCL) CXCL10) [54,57] (Figure 3a);
- The transneuronal spreading or “neuronal route” (via exocytosis/endocytosis or “fast axonal transport” mechanisms of vesicles along the microtubules track in order to move the virus from synaptic terminals back towards neuronal cell bodies) from systemic organs to the CNS throughout the cranial nerves: In this process, the virus first enters the nerve endings (i.e., the peripheral nerves) and then is retrogradely transported to the soma to invade the CNS; in detail, SARS-CoV-2 enters through (i) the olfactory mucosa (causing anosmia), and it spreads via the olfactory nerve to the olfactory cortex; (ii) the lacrimal and salivary glands, and it spreads via the facial VII and glossopharyngeal IX nerves to their respective brainstem nuclei; (iii) the taste buds of gustatory mucosa (triggering ageusia), and it spreads via the VII and IX nerves to the Nucleus Tractus Solitarius (NTS) located in the brainstem; or (iv) the respiratory system, and, via the vagus nerve X, it spreads both to other systemic organs (the heart, kidneys and gastrointestinal tract) innervated by this nerve and to the brainstem [58] (Figure 3b);
- The circumventricular organs (CVO) lacking the BBB: SARS-CoV-2 enters the CNS through the ACE2-expressing and vascularized subfornical organ, the paraventricular nucleus, the NTS and the rostral ventrolateral medulla by triggering local neurovascular damage (Figure 3b);
- The ocular system: the epithelial cells of the cornea and conjunctiva, the trabecular meshwork, choroid and retinal cells, optic nerve and geniculo-calcarin tract expressing the ACE2 receptor and neuropilin-1 are also entry points for the SARS-CoV-2 infection towards the occipital cortical areas [26,30] (Figure 3c).

Once in the CNS, as with other viruses endowed with neurotrophic properties, SARS-CoV-2 binds to neurons, astrocytes, oligodendrocytes and microglia, which all express, on their membrane, both ACE2 and TMPRSS2 receptors, and then spreads to multiple brain areas including the cerebral cortex, caudate/putamen, ventral striatum, thalamus, hypothalamus (paraventricular nuclei), spinal cord, hippocampus, frontal cortex, substantia nigra, middle temporal gyrus and along the synapse-interconnected anatomic networks, causing, eventually, neuronal cell dysfunction and degeneration [29,30]. 

## 3. Bidirectional Relationships between Long COVID-19 and AD

Severe and debilitating neurological complications that are classically associated with AD symptomatology, such as memory deficits (73%) and cognitive impairments (brain fog) (85%), have also been recorded in follow-ups more than 2 years after the resolution of the acute infection of SARS-CoV-2, occurring at similar rates in hospitalized and non-hospitalized adults [8,9,59,60,61,62]. In line with this finding, a growing body of longitudinal, prospective and retrospective studies indicates that the virus neurotropism can *per se* significantly trigger and/or contribute to the occurrence of AD-like neuropathological features in the brain, even though how the post-infection sequelae of COVID-19 actually impacts mental processes concerning the acquisition, storage, integration and retrieval of information needs to be fully clarified [30,63,64,65,66,67,68]. In addition, whether the reciprocal association between SARS-CoV-2 and AD implies a direct causal relationship and/or originates from chronic and excessive systemic inflammatory conditions also remains to be determined. Nevertheless, a strong bidirectional relationship existing between the COVID-19 infection and AD development has been clearly documented [69,70]. On one side, elderly individuals with AD are more prone to the SARS-CoV-2 infection, showing an increased chance of severe COVID-19 complications and mortality [71,72,73,74,75,76,77]. On the other side, people who have experienced COVID-19 are at a greater risk of suffering AD, with a global reduction in attention and executive and visuospatial functions [77,78,79,80,81,82,83,84,85,86,87,88,89,90]. Consistently, a marked mental decline in connection with an overall reduction in brain size and a diminution of grey matter thickness in the orbitofrontal cortex and para-hippocampal gyrus—two cerebral areas that are largely affected in AD subjects—have been demonstrated in subjects recovering from COVID-19 when compared to healthy controls, even in non-hospitalized patients [91]. In addition, a mouse model of mild SARS-CoV-2 infection displayed several morphological, molecular and biochemical markers typical of AD, including an impaired hippocampal neurogenesis, microgliosis, myelin disintegration, elevated CSF levels of cytokines/chemokines, such as CCL11, and neuronal loss with cognitive dysfunctions [92]. Bioinformatic screening of the SARS-CoV-2 proteome has also revealed different peptides with a high propensity to self-aggregate into amorphous and fibrillary amyloid clumps which are toxic to neurons, which also occurs in AD brains [93]. Anosmia, due to sustained and protracted inflammation, is caused by the persistence of the SARS-CoV-2 virus in the olfactory mucosa and/or in the olfactory bulb of the COVID-19-affected brain [94,95,96,97,98,99], and, in parallel, this sensorial complication, particularly the inability of olfactory identification/discrimination, is visible in the early/prodromal stages of AD subjects suffering from Mild Cognitive Impairment (MCI) [100]. Hypometabolism detected in AD brains with BBB leakage/dysfunction and cerebral microvascular changes have been also reported in patients with long COVID-19 in correlation with specific cognitive symptoms [101,102]. Furthermore, the activation of Kynurenine signaling—a cellular pathway whose stimulation is involved in the regulation of immune tolerance, neurotoxicity and vascular injury—is dysregulated both in AD [103] and COVID-19. To this point, in a large cohort of cases recovering from mild-moderate to acute SARS-CoV-2 infection across a 12-month period, a causal relationship among the presence of its typical metabolites, such as Quinolinic Acid (QA) and Kynurenine (Kyn) 3-HydroxyKynurenine (3HK) and 3-hydroxyantranilic acid (3HAA), intellectual disabilities and anosmia has been recently reported [104]. In the reminiscence of structural and metabolic mitochondrial alterations responsible of energy deficiency that drives the loss of dendritic spines and synapses occurring in AD development [105,106], abnormal levels of mitochondrial proteins as well as SARS-CoV-2 spikes and nucleocapsid proteins have been also detected both in neuron- and astrocyte-derived exosomes in the plasma of COVID-19 patients with neurological and psychiatric manifestations [107]. More importantly, the SARS-CoV-2 infection provokes and/or precipitates several neurodegenerative processes, in particular, widespread neuroinflammatory response, synaptic pruning, protein misfolding, the disruption of the oxidation-reduction systems, damage to blood vessels by coagulopathy and endothelial dysfunction and neuronal injuries, that are all traits classically discernable in AD brains [108]. As a matter of fact, the virus damages not only the post-mitotic neurons but also the surrounding astrocytes and microglia and, thus, indirectly further aggravates the brain injury, owing to the exaggerated release of pro-inflammatory cytokines and/or deleterious Reactive Oxygen Species (ROS) [32]. Moreover, in addition to triggering neurodegeneration and neuroinflammation, SARS-CoV-2 also promotes the chronicity of these changes, up to months or even years after the acute infection, since it invades and diffusely infiltrates/propagates throughout the brain via trans-synaptic spreading along the motor-based, microtubule-dependent axonal transport [26,31,51,66]. More importantly, ACE2 is co-expressed in both GLUtamatergic and GABAergic neurons, indicating that, in the CNS, SARS-CoV-2 infection is able to interfere with the signaling transduction pathways activated by these two neurotransmitters regulating the cortical excitability. Therefore, SARS-CoV-2 seems to initiate and/or exacerbate the imbalance between excitatory and inhibitory electrical neuronal circuits, leading to excitotoxicity and cell loss, which also occurs in AD progression [109,110]. Finally, based on the evidence connecting the repeated infection of Herpex Simplex Virus type-1 (HSV-1) and amyloidosis, the viral re-activation of SARS-CoV-2 in the CNS in concomitance with an age-dependent physiological decline of innate immunity is more likely to trigger an inflammatory process which, in turn, increases the Aβ synthesis and accumulation, as well as the hyperphosphorylation of tau (pTau) and aggregation, a cascade that is suggestive of the so-called “infection hypotesis of AD” [68,111]. 

## 4. Common Risk Factors and Involved Mechanisms That Mediate the Association between COVID-19 and AD 

**Figure 4:**
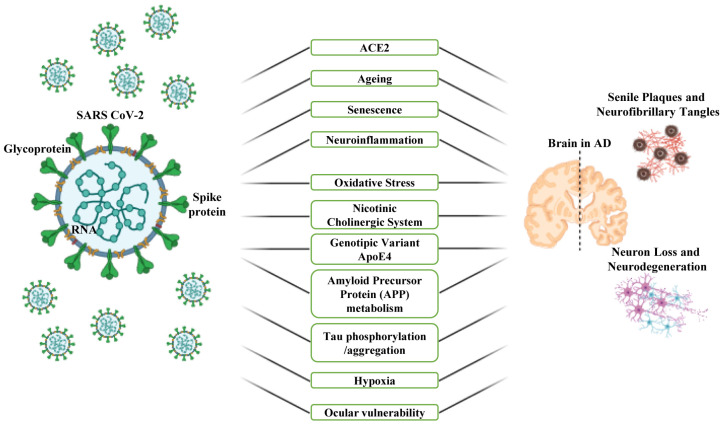
Common mutual risks and pathophysiological mechanisms in the COVID-19 pandemic and Alzheimer’s Disease (AD). A schematic of key risk factors associating the COVID-19 and AD pathogenesis. This figure was created with BioRender.com.

Compelling studies have shown that COVID-19 and AD share several physiopathological aspects including ACE2 expression, age, inflammation with “cytokine storm”, oxidative stress, the APOE4 genetic variant, the neurotransmitter system, hypoxia and the activation of intracellular pathways associated with the altered metabolism of APP/Aβ and tau (Figure 4). 

### 4.1. ACE2 and Ageing 

ACE2, the SARS-CoV-2 receptor required for cell entry, is considered the most important determinant in dictating the greater susceptibility of developing AD among COVID-19 patients [108,112]. Microarray, Western blotting, Reverse Transcription quantitative Polymerase Chain Reaction (RT-qPCR) and immunostaining analyses have undoubtedly shown that the expression levels of ACE2 significantly increase in the brain tissues of human AD subjects when compared with healthy, not-demented controls and in close relationship with the severity of clinical dementia and different neuropathological parameters, including the density of dystrophic neurites, the Aβ plaques and NFT accumulation [113,114,115]. In addition, in a SARS-CoV-2 pseudovirus infection model, the fibrillogenic and highly-neurotoxic Aβ1-42 peptide—but not the shorter Aβ1-40 one—binds to both the S1 protein and ACE2 receptor [116], by facilitating the virus invasion and production of IL-6. Apart from being a SARS-CoV-2 receptor, ACE2 is also a key regulator of the Renin-Angiotensin System (RAS) that is one of the most complex hormonal regulatory axes involved in maintaining the body homeostasis and exerting a broad range of other important functions in multiple organs, in particular the cardiovascular and immune systems. In detail, ACE2 catalyzes the Angiotensin II conversion to Angiotensin(1–7) (Ang 1–7) which, in turn, binds to its G Protein-Coupled Receptor (GPCR) MAS to regulate several downstream signaling cascades, for instance the Phosphatidyl-Inositol 3-Kinase (PI3K)/Akt serine/threonine kinase 1 (Akt/) cAMP response element-binding protein(CREB)/Brain-Derived-Neurotrophic Factor (BDNF)/Tropomyosin receptor kinase B (TrkB) [112]. Interestingly, the SARS-CoV-2 infection seems to instigate and/or accelerate the AD phenotype by inhibiting ACE2 enzymatic activity, triggering a hyperinflammatory response and downregulating the secretion of BDNF, a potent neurotrophin endowed with crucial functions in supporting neurogenesis, cognition and the prevention of neurodegeneration upon binding to its cognate transmembrane TrkB receptor protein [117]. In concomitance with an elevation in the mRNA transcript of ACE2 facilitating the entry points of SARS-CoV-2 in the CNS, a high level of its TBS/Detergent-soluble inactive form has also been detected in the parietal cortex of two large cohorts of AD-fully diagnosed subjects when compared to controls, suggesting that a defective brain RAS signaling with a consequent decrease in its anti-inflammatory and neuroprotective properties is more likely to take place in humans with a low cognitive score [115].

Ageing is the greatest contributing factor to AD onset/progression by causing genomic instability, telomere shortening, epigenetic modifications, a loss of proteostasis, a decline in mitochondrial respiration and energy production, deregulated nutrient sensing, altered intercellular communication, an increased permeability of BBB and deregulated inflammation [118]. Another important hallmark of ageing is cellular senescence [119,120], a terminal state of cell-cycle arrest characterized by the proinflammatory Senescence-Associated Secretory Phenotype (SASP) as result of an increased release of various tissue-remodeling (e.g., Tumor Growth Factor (TGF) TGF-β and MMPs) and immune-related (e.g., IL-6, IL-8 and IFNs) factors involved in regeneration/repair and immunosurveillance. In chronic age-associated neurodegenerative diseases such as AD, these events persist for long time and then turn out to be detrimental, with consequent organ dysfunctions, aberrant paracrine senescence and chronic inflammation [121,122]. In line with this notion, the elimination of senescent cells by means of senolytic compounds significantly mitigates the extent of neuropathology [123]. It is noteworthy that SARS-CoV-2 infection can induce *per se* a condition of premature senescence both directly, by increasing the secretion of InterFeroNs (IFNs) and other pro-inflammatory mediators such as CXCL-10, CCL-2, IL-6, IL-8, IL-12, IL-1β, IFN-γ and TNF-α from infected cells, and/or indirectly, by promoting the release of Danger-Associated-Molecular Patterns (DAMPs) via necroptosis and pyroptosis [124,125]. Moreover, and more importantly, older COVID-19 patients are more likely to accumulate huge levels of cellular senescence, since aged tissues show a decreased intrinsic capacity of repairing damages and/or eliminating senescent cells via the immune system [126]. Relevantly, the age-dependent decay of immune defense against SARS-CoV-2 infection, the so called “immunosenescence and inflamm-aging”, plays a major role in boosting the vulnerability to severe COVID-19 outcomes in older adults [127,128,129,130]. In agreement, in COVID-19 patients, a strong association has been documented between the severity of infection with more severe-to-lethal outcomes and the presence of the immunosenescence phenotype with a high level of the Neutrophils-to-Lymphocytes Ratio (NLR) [131] and IL-6 production [132]. Interestingly, the reduction in telomere elongation and the reactivation of reverse transcriptase telomerase [133], two important molecular hallmarks of cellular senescence in chronic neurodegenerative diseases, critically influence the severity of COVID-19 symptoms, as proved by the observation that an elevated risk of developing grave and fatal complications is found in SARS-CoV-2-infected patients carrying shorter telomeres from their peripheral blood lymphocytes [134]. Several senolytic compounds, such as the flavonoid Quercetin and the mammalian Target of Rapamycin (mTOR) kinase inhibitor Sirolimus—both known to reduce the SASP and prevent the senescence induction (geroconversion)—are currently exploited in clinical trials to counteract the long-COVID-19 syndrome [126]. Finally, the up-regulation of the steady-state expression level of ACE2 occurring in several human tissues with an increasing age, mainly in the nasal neuroepithelium, which is one of the most accessible routes of SARS-CoV-2 invasion in the CNS, also accounts for the elevated risk of contracting COVID-19 in the elderly population in connection with poor clinical outcomes [135]. 

### 4.2. Neuroinflammation, Oxidative Stress and Nicotinic Cholinergic System

Apart from the premature synapses’ elimination and neuronal deterioration tightly associated with a cognitive decline, a pronounced neuroinflammation characterized by reactive microglia, astrogliosis and the infiltration of cytotoxic CD8-positive T cells is among the most prominent neuropathological traits discernable in the brains of patients who died from both AD [136,137,138,139] and COVID-19 [37,140,141]. In this regard, COVID-19 chronic inflammation is caused both directly, by the SARS-CoV-2 infection of the CNS, and indirectly, by peripheral inflammation via immune-to-brain signaling [52]. Consistently, IL-6, IL-1, TNFα, complement proteins and Galectin-3/9 are common prognostic biomarkers for the activation of inflammatory immune responses in the CNS, following both SARS-CoV-2 neuroinvasion and AD [142]. By single-nucleus RNA sequencing (snRNA-seq), followed by immunohistochemical staining validation, an excessive stimulation of microglia and brain-barrier inflammatory signals in concomitance with a downregulation in the expression of neuronal genes encoding several synaptic vesicle components, such as synaptobrevins (VAMP1 and VAMP2), SynTaXin 1B (STX1B) and the SyNAPtosome-associated protein of 25 kDa (SNAP25), which regulate the glutamate release and excitatory neurotransmission, have been also documented in post-mortem brain tissues from individuals with AD [143] and COVID-19 (frontal cortex and choroid plexus) [144]. Moreover, the activation of PYrin (PYD)-Domain-containing protein 3 inflammasome (NLRP3) [145], which affects the microglial-dependent clearance of Aβ [146] and promotes tau pathology [147], is triggered in the brain as a consequence of SARS-CoV-2 neuroinvasion, just as described in the AD etiology. 

Oxidative stress with the excessive production of the harmful ROS provoking Aβ accumulation/aggregation and tau hyperphosphorylation in AD brains [148] also takes part in the innate response against SARS-CoV-2 invasion [149,150]. A large amount of activated radical-producing neutrophils are found in COVID-19 patients, consistent with a massive production of ROS [151,152]. Changes in mitochondrial respiration and associated redox imbalances have been detected in Peripheral Blood Mononuclear Cells (PBMCs) from patients with COVID-19, in agreement with the energy supply required for the production of pro-inflammatory cytokines during the virus-triggered immune response [153]. Interestingly, upon exposure to SARS-CoV-2, the activation of inducible Nitric Oxide Synthase (iNOS), an important biological mediator of inflammation and immunoregulation producing Nitric Oxide (NO) from L-arginine, causes an overproduction of the superoxide radical ion (O_2_^−^) in people who have survived the acute phase of COVID-19 that becomes self-perpetuating, even when the virus has been cleared, turning into a persistent and protracted free radical-induced damage [154].

In addition to being involved in the CNS in high-order cognitive processing, sensory information integration, sleep and wakefulness, Acetylcholine (Ach) and its nicotinic Receptors (nAChRs) play a pivotal role in the homeostatic regulation of inflammatory response, owing to the high expression of the α7 receptor (α7nAChR) on the surface of immune cells (B cells, macrophages and T cells) [155,156,157]. Therefore, it is not surprising that the dysregulation of the nicotinic cholinergic system is involved, in parallel, in both COVID-19 and AD pathophysiology [158,159,160]. In support of this finding, the SARS-CoV-2 S1 glycoprotein protein has proved to interact with the cholinergic nicotinic ACh receptor α7 (α7nAChR) and to negatively impair its function by preventing the acetylcholine’s binding and, in turn, the specific intracellular activation of its downstream signaling(s) [161,162,163]. Interestingly, nicotine—a selective agonist of α7nAChR—was recently approved as a promising therapeutic option to counteract the neurological complications in COVID-19 syndrome, as a direct result of its potent anti-inflammatory and neuroprotective actions [159,164]. Likewise, the neocortical cholinergic innervation is also gradually destroyed in AD brains—mainly due to the sequential and aberrant deposition of the insoluble, ThioflavinT (ThT)-positive, dense SP and NFTs—with consequent clinical changes in cognition, behavior, mood and emotions [160]. In particular, alterations in acetylcholine release in concomitance with a decrease in high-affinity choline uptake and a downregulation in muscarinic and nicotinic acetylcholine receptor expressions represent a solid ground for the cholinergic hypofunction detected in AD. In line with this so-called “cholinergic hypothesis”, in vitro and in vivo studies have clearly shown that the Low Molecular Weight (LMW) oligomeric Aβ conformers—which are the most neurotoxic species to synapses found in autoptic AD brains—actually bind to and impair the function of the α7nAChR, both in cultured hippocampal neurons and in mouse models [158,159,160]. Finally, similar to COVID-19, a pharmacological strategy in the clinical management of AD is based on the preservation/restoration of cholinergic neurotransmission, and, in this framework, several FDA-approved cholinesterase inhibitors such as donepezil, rivastigmine and galantamine are currently used in therapy to slow down the cognitive and functional decline in affected patients [158,159,160]. 

### 4.3. APOE Genetic Variant and Signal Pathways

Another strong risk factor which accounts for more than 95% of all sporadic AD cases is the genetic variant APOE4 [164,165,166] that plays a causal role in the alteration of cellular trafficking/the metabolism of cholesterol and in the regulation of the aggregation state and deposition of Aβ peptide(s). Interestingly, the APOE polymorphism is also involved in the COVID-19 syndrome by increasing the incidence and severity of the SARS-CoV-2 infection and neurodegeneration [167,168,169,170,171,172,173,174]. In this connection, several lines of evidence have shown that the APOE4 genotype (i) increases the permeability of the BBB, which makes patients more susceptible to viral infections [175]; (ii) augments the production of pro-inflammatory cytokines by peripheral macrophages and CNS microglia [176]; (iii) elevates the infectivity of SARS-CoV-2 both in neurons and astrocytes [177]; (iv) controls the cholesterol homeostasis which, in turn, facilitates the binding of the S1 protein to the ACE2 receptor during the first step of the SARS-CoV-2 infection [178]; (v) decreases the expression of several antiviral defense genes, including InterFeron-Induced TransMembrane Proteins (IFITM) IFITM2 and IFITM3, InterFeroN Alpha(α) and Beta(β) Receptor Subunit 1 (IFNAR1) and Lymphocyte Antigen 6 Family Member E (LY6E) [179,180,181]; and (vi) downregulates the ACE2 expression, followed by an imbalance in Renin-Angiotensin System (RAS) that catalyzes the degradation of Angiotensin II (Ang II) to Angiotensin(1–7) (Ang 1–7), associated with the chronic hyperinflammatory state of COVID-19 [182].

The activation of intracellular signaling transduction pathways associated with APP/Aβ and tau pathologies represents an additional relevant commonality, putting in mutual relation the occurrence of COVID-19 and AD. It is widely acknowledged that the main histopathological features detected in the vulnerable regions of AD brains, such as the entorhinal region and the hippocampus which are involved in memory/learning and synaptic plasticity, are the SP and the NFTs. These two lesions are, respectively, composed in their aggregated forms of Aβ peptide(s)—generated by the aberrant and sequential beta/gamma (β/γ)-mediated amyloidogenic proteolysis of its membrane precursor Amyloid Precursor Protein (APP) involved in synaptogenesis and neurogenesis—and by abnormally hyperphosphorylated and/or truncated tau protein, whose function is the modulation of the intracellular stability of axonal microtubules [183,184]. Consistently, the mRNA of the holoprotein APP has turned out to be greatly upregulated in single-cell RNA-seq studies carried out on blood samples from COVID-19 survivors in comparison with controls ones [185] and on oligodendrocytes isolated from their post-mortem brain tissues [186], hinting at the deregulation of APP metabolism and its proteolytic cleavage. Furthermore, just as detected in AD, a tendency toward an accelerated APP amyloidogenic processing/Aβ deposition in the brains from patients with the COVID-19 neurological syndrome is evident when compared to healthy controls and in connection with significant lower amounts of the soluble Amyloid Precursor Protein alpha and beta fragments (sAPPα and sAPPβ) as well as the Aβ40, Aβ42 and Aβ42/Aβ40 ratio in their peripheral CSFs [187]. The transcriptomic and interactomic profiles from the frontal cortex of fully diagnosed AD subjects with cognitive decline who have also experienced COVID-19 have revealed that SARS-CoV-2 can indirectly amplify the Aβ toxicity in the brain, giving rise to neuroinflammation and an imbalance in the relative levels of cellular pro-oxidants and antioxidants [188]. 

Relevantly, Aβ1-42, but not Aβ1-40, binds with high affinity to both the viral S1 protein and ACE2 receptor of SARS-CoV-2 [189]. Immunohistochemical staining studies using different specific antibodies clearly decorate insoluble, proteinaceous Aβ-positive aggregates in the autopsied brains of patients who died of COVID-19 [190]. From a mechanistic point of view, immunofluorescence and RNA-seq analyses performed on the cortical and hippocampal tissues of transgenic mice expressing human Angiotensin-Converting Enzyme 2 (hACE2) suggest novel insights by showing, for the first time, that the SARS-CoV-2 spike protein S2 subunit is able *per se* to enhance the Aβ production via direct binding to and the modulation of the processing enzymatic activity of the γ-secretase complex [191]. A persistent brain neuropathology with the accumulation of AT8 (ptauSer202, Thr205), an AD-pathognomonic site of tau hyperphosphorylation, has been also reported in Syrian golden hamsters after the intranasal inoculation of SARS-CoV-2 [192]. Furthermore, the activation of NF-κB signaling driving the neuroinflammatory cascade in response of SARS-CoV-2 neurotropism [193,194] also activates the beta(β)-site APP cleaving enzyme 1 (BACE-1) activity, thereby triggering the first step of proteolytic cleavages sub-serving the sequential generation of Aβ peptide(s) [195]. A marked elevation in tau phosphorylation at multiple AD-like epitopes, such as pSer262, pSer214 and pSer356 and pSer199/202, has also been ascertained in COVID-19 brains in concomitance with the activation of several known tau-directed kinase, including the AMP-activated Protein Kinase (AMPK), Glycogen Synthase Kinase 3 beta (GSK3β), Protein Kinase A (PKA) and Calcium/Calmodulin-Dependent Protein Kinase II (CAMKII) [196]. Moreover, the levels of specific peripheral biomarkers that are routinely used in clinical practice for AD diagnosis—such as total tau protein in association with IL-6, Neurofilament Light Chain (NFL) and Glial Fibrillary Acidic Protein (GFAP)—are elevated in the CSF and serum of COVID-19 patients [197,198]. More importantly, SARS-CoV-2 targets the neurons of 3D human brain organoids, inducing an aberrant subcellular redistribution of tau from axonal processes to soma, hyperphosphorylation at Threonine 231 (Thr231), apoptotic caspase-3 activation and, eventually, neuronal death, specific cellular changes that are reminiscent of key features associated with AD neuropathology [199]. In a similar way, hyperphosphorylation at the Ser262 and Ser396 sites and the mislocalization and increased aggregation of tau are also detected in human neuron-like SH-SY5Y cells after in vitro infection with different clinical strains (B.1, B.1.1.7 and B.1.617.2) of SARS-CoV-2 [200]. Furthermore, by using a combination of ThT assay, Transmission Electron Microscopy (TEM) staining, analytical High-Performance Liquid Chromatography (HPLC) and Mass Spectrometry (MS), Eberle and colleagues have recently reported that tau is proteolytically cleaved in vitro by the viral SARS-CoV-2 3CL protease with a consequent release of the 25kDa fragment, triggering the formation of amorphous fibrils, resembling the paired helical (PHF) and/or straight filaments (SFs) typically detected in AD brains [201]. Finally, in brain cortices from Murine Hepatitis Virus-1 (MHV-1) coronavirus-inoculated mice—an in vivo model which is very similar to the SARS-CoV-2 infection observed in humans—a significant increase in AT8-tau hyperphosphorylation along with reactive astrocytes and microglia (GFAP and Iba1-positive, respectively) and reduced synaptophysin-1 synaptic protein are found up to 12 months post-infection, again recapitulating several characteristic features of chronic neurodegenerative human tauopathies, including AD [202]. 

### 4.4. Hypoxia

Among the major clinical manifestations of COVID-19 with acute respiratory distress syndrome (ARSD) stands out hypoxia, a pathological condition that is caused by the lack of oxygen and/or the accumulation of mitochondrial ROS into the lungs’ upper airways due to a maladaptive inflammatory response to viral penetration [203]. Relevantly, the SARS-CoV-2 infection induces the cellular expression of Hypoxia-Inducible Factor 1α (HIF-1α), an important transcriptional factor responsible for cellular adaption to low oxygen tension, which, in turn, plays a key role in driving the virus-mediated inflammatory response (cytokines storm), metabolic reprogramming and oxidative stress [204,205,206]. Just as SARS-CoV-2 damages the pulmonary tissue locally inhibiting the gas exchange, post-mitotic neurons with high energy requirements are particularly vulnerable, in the brain, to any subtle change in oxygen saturation, resulting in the activation of pro-apoptotic signaling pathways and, eventually, neuronal injuries [203]. Therefore, it is not surprising that hypoxia significantly increases the risk of developing neurodegenerative diseases, in particular AD, with the dysregulation of the HIF-1α pathway leading to AβPP amyloidogenic processing with Aβ accumulation, due to increased production [207,208,209] and/or decreased degradation [210], tau hyperphosphorylation and microglial activation [211]. Focal deposits of Aβ have been detected in the brains of young (less than 60 years old) hospitalized patients who died of COVID-19 in correlation with widespread hypoxic damage [212]. Interestingly, the important contribution of oxygen dyshomeostasis to long-term cognitive impairment in the post-COVID-19 syndrome has been highlighted in a recent follow-up study reporting that an improved memory and attentional capacities, likely due to delayed hippocampal damage, are observed in a small cohort of patients that underwent oxygen therapy and were prospectively recruited after 3–9 months of the SARS-CoV-2 infection [213]. In line with this, prolonged hyperbaric oxygen treatment can reduce hypoxia, neuroinflammation, the accumulation of Aβ and phosphorylated tau, leading to a significant improvement of cognitive performances in a 3xTg AD mouse model carrying three mutations associated with familial AD (APP Swedish, MAPT P301L, and PSEN1 M146V) when tested in hippocampal-dependent behavioral tasks [214] and, possibly, in affected patients [215].

### 4.5. Serotonin or 5-Hydroxytryptamine (5-HT)

Serotonin or 5-HydroxyTryptamine (5-HT), a monoamine neurotransmitter involved in the control of mood/reward and learning, is an additional link to explaining the neurocognitive impairments associated with COVID-19 and AD. In this regard, an elegant study recently reported that the long COVID syndrome is associated with a low peripheral level of circulating serotonin which, in turn, impairs the hippocampal-dependent memory function via the reduced stimulation of the vagus nerve signaling [216]. Interestingly, Selective Serotonin Reuptake Inhibitors (SSRIs) are widely prescribed to treat neurobehavioral symptoms associated with dementia [217], and lower levels of serotonin have been detected in AD brains [218]. 

## 5. Neuro-Ophthalmic Complications Shared by COVID-19 and AD

Compelling experimental, molecular, histological and clinical studies suggest that the neuro-ophthalmic system and related visual manifestations are another important pathogenetic connection between the COVID-19 and AD syndromes (Figure 4). 

In this context, the eye and associated ocular structures are possible transmission routes of SARS-CoV-2 penetration to the brain [219,220,221,222,223,224]. Moreover, in addition to the visual pathway which provides a direct anatomical connection between the ocular surface and the brain, the hematogenous route has been recently proposed as an alternative mode of penetration/transmission of the virus from eye to body. Indeed, after the infection of the iris and conjunctival cells both expressing the ACE2 receptor, SARS-CoV-2 can reach the blood capillaries and then gain access through the Blood–Retinal Barrier (BRB) in the Retinal Pigment Epithelium (RPE) and blood vessel endothelial cells to reach, eventually, the bloodstream and infect the extraocular areas [220]. Thus, in humans, the localization of the ACE2 receptor, required for an efficient viral entry from the eyes, can be considered both intra- and extra-ocular, with large expressions in conjunctival and corneal cells, retinas and retinal pigment epithelium [224,225,226,227,228,229]. In agreement, SARS-CoV-2 can infect and replicate in retinal organoids, and quantitative Real-Time Polymerase Chain Reaction (RT-PCR) analyses have confirmed the presence of SARS-CoV-2 genomic RNA in different ocular tissues including human retina, cornea, conjunctiva, lacrimal sacs and tears from deceased cases with COVID-19 [230,231,232,233,234]. Finally, ocular complications are frequently described by patients both during and after recovery from COVID-19, especially conjunctivitis, retinopathy (retinitis, retinal hemorrhages, retinal venous and arterial occlusion), uveitis, vitritis, optic neuritis [219,222,235,236,237,238,239,240] in association with signs of excessive inflammation, nerve fiber loss, increased dendritic cell density, impaired retinal microcirculation and poor vision [239,241,242,243,244,245,246]. 

Regarding AD, there is a growing body of literature endorsing the concept that the two hallmarks classically discernable in the brains of affected patients and preclinical animal models—i.e., the deposits of Aβ and hyperphosphorylated tau protein—are also present in their eyes, sometimes even before the appearance of clinical cognitive symptoms, in close association with other ocular pathophysiological alterations such as nerve fiber layer thinning, the degeneration of retinal ganglion cells, vascular alterations, local inflammatory responses and gliosis [247,248,249,250,251,252]. These findings are in agreement with the fact that the retina and optic nerve are neurodevelopmental outgrowths of the CNS, while the aqueous humor and tear film located in the anterior eye segment are more likely to be comparable to the CSF. Consistently, changes in functional visual processing are detected in subjects suffering from AD, including a loss of the visual field, decreased contrast sensitivity, low visual acuity, impaired color vision or motion perception and visuospatial deficits [253,254,255,256,257,258,259,260]. More importantly, in light of the great accessibility of the eyes, which are considered a direct “window” to brain, advanced high-resolution imaging techniques detecting ocular Aβ and pTau in the retina are currently used as predictive and diagnostic biomarkers in the clinical management of AD by allowing for the large-scale noninvasive screening and monitoring of at-risk populations [246,261,262].

## 6. Conclusions and Future Perspectives

Despite the great interest in the COVID-19 pandemic outbreak and its neurological consequences, it is important to remember that there are still several controversial results concerning the presence of the SARS-CoV-2 virus in the brain [263]. To this point, several studies have identified the direct neuro-invasive capacity of SARS-CoV-2 to enter the brain [264,265] while others do not confirm the presence of the virus within the brain [38,266,267] or report very low levels of detectable RNA and viral protein brains [37,140], suggesting that the virus neuropathology is more likely to be mediated by cytokines through systemic effects [52].

Nevertheless, among the long-term manifestations of post-COVID-19, AD-like dementia stands out as the most frequent disorder with higher susceptibility of subjects exposed to the SARS-CoV-2 infection toward more severe clinical outcomes [268]. Relevantly, even though COVID-19 and AD have different clinical presentations, there are multiple, neurological, psychiatric and ophthalmological, physiopathological aspects linking with each other and increasing the patients’ complications and mortality. 

The cellular mechanisms underlying the COVID-19-induced cognitive impairment and visual deficits mainly include the SARS-CoV-2 neurotropism to the CNS and the eyes as a potential route of the virus’s invasion of the brain [269,270]. In addition, several common risk factors such as excessive neuroinflammation, ACE2 expression, APOE4 genotype, age, oxidative stress, hypoxia, neurotransmitter system, the activation of signaling pathways associated with APP/Aβ and tau pathologies provide solid neurobiochemical correlates for reciprocal associations between COVID-19 and AD [271]. In addition, the “inflamm-aging” not only predisposes one to the SARS-CoV-2 infection but also reduces the antibody response to vaccinations, reinforcing COVID-19 as a risk factor in developing cognitive impairments and dementia in frail and elderly patients [272,273]. Clinical follow-up studies with the intent of evaluating the extent and the duration of cognitive impairment in large cohorts of COVID-19 patients along with further experimental investigations on SARS-CoV-2-infected human brain organoids possibly recapitulating the phenotypic expression of key AD hallmarks are still needed. From a translational point of view, the concerted effort from clinicians, researchers, patients, caregivers and health and social care agencies, in association with a deep understanding of the biological aspects linking COVD-19 and AD physiopathologies, will help in designing specific diagnostic/therapeutic strategies [274,275], in order to mitigate the impact of long-lasting neurological and ophthalmological COVID-19 complications in the aging population.
